# Bilateral Page kidney in a postpartum female: a case report

**DOI:** 10.1097/MS9.0000000000000196

**Published:** 2023-02-07

**Authors:** Pradeep Regmi, Suraj Shrestha, Elisha Poddar, Deewakar Sharma

**Affiliations:** a Department of Radiology, Tribhuvan University Teaching Hospital; b Maharajgunj Medical Campus, Institute of Medicine; c Department of Cardiology, HAMS Hospital, Kathmandu, Nepal

**Keywords:** ARB, Page kidney, percutaneous drainage, subcapsular hematoma

## Abstract

Page kidney results from external compression of the kidney by a subcapsular hematoma and is one of the rare but treatable causes of secondary hypertension. The majority are traumatic or iatrogenic and often unilateral. Spontaneous bilateral Page kidney is rare.

**Case Presentation:**

A 35-year P1 with gestational hypertension presented with a persistent postpartum elevation of blood pressure (BP). Imaging studies revealed bilateral renal subcapsular hematoma (left>right). She was managed with an angiotensin receptor blocker initially and ultrasound-guided percutaneous drainage of the collection for the optimal control of elevated BP.

**Clinical Discussion:**

Ultrasonography and computed tomography of the kidneys are the most frequently used for diagnosing a Page kidney. Medical management with antihypertensive and regular follow-ups form the first line of treatment in Page kidneys. Percutaneous drainage, surgical decortication, laparoscopic intervention, and nephrectomy are necessary in cases of organized late hematomas.

**Conclusion:**

Spontaneous bilateral Page kidney is a rare but potentially treatable and curable form of hypertension. Percutaneous drainage is an effective method to drain the hematoma and control elevated BP.

Highlights

- Page kidney results from compression of the kidney by subcapsular hematoma.
- Spontaneous bilateral Page kidney is very uncommon.
- Percutaneous drainage of hematoma can help attain optimal blood pressure.

## Introduction

Page kidney or Page phenomenon is a rare cause of secondary hypertension due to external compression of the kidney by a subcapsular hematoma. It is an uncommon cause of secondary hypertension from resulting renin–angiotensin–aldosterone system activation^1^. Though various etiologies are responsible for this entity, blunt trauma is the most common cause^2^.

In addition, iatrogenic causes including medical interventional procedures such as renal biopsy, extracorporeal shock wave lithotripsy, and percutaneous antegrade endopyelotomy can cause subcapsular hematoma leading to a Page kidney^3^,^4^.

Nontraumatic or spontaneous causes of Page kidney are uncommon with isolated cases reported in the literature. These include spontaneous renal hemorrhage secondary to underlying tumors, arteriovenous malformations, cyst rupture, glomerulonephritis, antiplatelet therapy, or vasculitis^5^,^6^. In some cases, Page kidney may be idiopathic^4^,^7^. Majority of cases are unilateral. Rarely, spontaneous bilateral Page kidney has been described due to metastasis of choriocarcinoma, antiplatelet drug therapy, and even during pregnancy^6^,^8^.

Herein, we report a case of spontaneous idiopathic bilateral Page kidney in a postpartum female who presented with persistently elevated blood pressure (BP). This case has been written in reported in line with SCARE criteria^9^.

## Case history

A 35-year-old female, 4 months postpartum period after her first pregnancy presented with a consistent rise in her BP which reached up to 180/100 mm Hg. The patient was diagnosed with gestational hypertension during her second trimester of pregnancy and was under antihypertensive medications during her pregnancy. She delivered a healthy-term baby via a lower-segment cesarean section without complications. However, her hypertension progressively increased and reached 180/100 mm Hg in her postpartum period. There was no history of headache, blurring of vision, decreased urine output, chest pain, or dizziness. In addition, she did not complain of high temperature, dysuria, other urological symptoms, or a history of trauma. There was no significant medical/surgical history.

**Figure 1:**
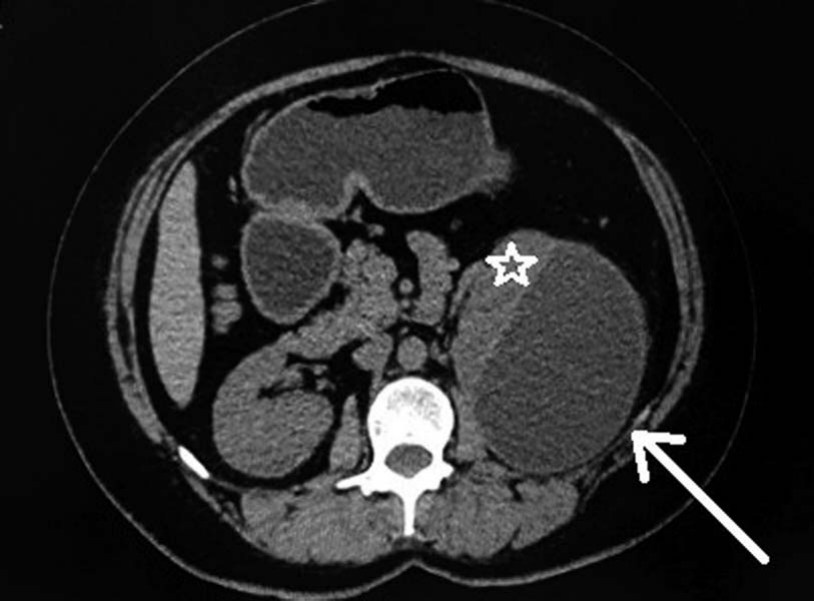
Axial noncontrast computed tomography shows hypodense collection in the subcapsular region of the left kidney (shown by arrowhead). The kidney appears compressed and displaced anteriorly (shown by a white star). The right kidney appears normal.

**Figure 2:**
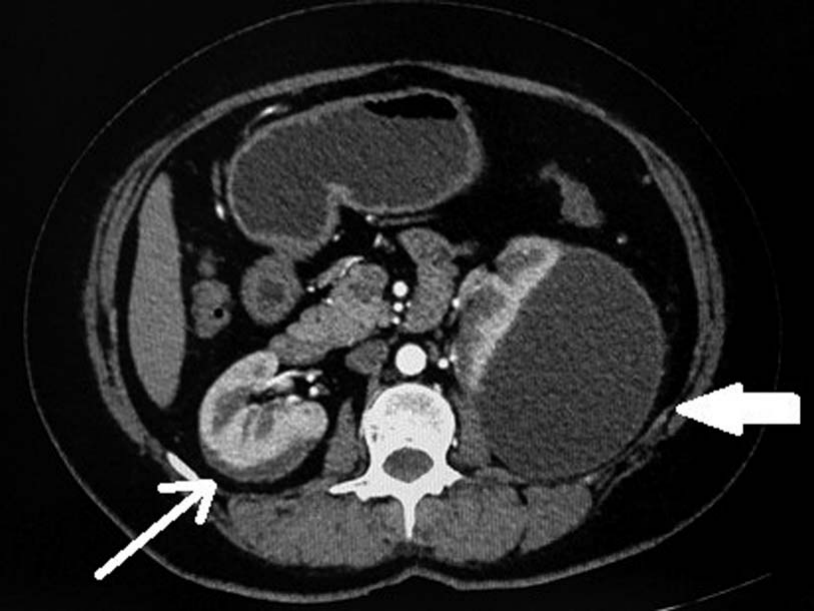
Axial contrast computed tomography shows a well-defined subcapsular collection with thin peripheral enhancement (shown by white arrow). The minimal subcapsular collection is also seen on the right kidney (shown by a thin white arrow).

Laboratory tests including a whole blood count and biochemical profile were normal. Prothrombin time/International normalized ratio and Bleeding time also were within normal limits. Serum creatinine was 1.2 g/dl. Due to a persistent rise in BP even during her postpartum period, she was advised for ultrasonography which revealed a large subcapsular collection in the left kidney of ~1 l which is compressing and displacing the left kidney. However, a mild increase in RI was seen in the Doppler of the renal artery without stenosis. Thus, the patient was advised for contrast-enhanced computed tomography (CT) abdomen for further evaluation On contrast-enhanced CT, a large hematoma was seen in the subcapsular region of the left kidney. The minimal subcapsular collection was also seen around the right kidney. No evidence of obvious active bleeder was seen in the arterial or venous phase. Delayed excretion of the contrast was seen from the left kidney. Bilateral ureters appeared normal with normal opacification in delayed phases. Bilateral suprarenal regions appeared normal (Figs 1, 2). The patient was prescribed losartan for a few weeks which could not control her BP to an optimal level. Thus, she underwent pigtail tube insertion by a team of interventional radiologists under local anesthesia which drained about 950 mL of blood. Her BP progressively fell down to 120/80 mm Hg and is currently doing well and is off medications.

## Discussion

Page kidney can also result when there is external compression of the kidneys due to a tumor, lymphocele, or urinoma, and subsequent renin–angiotensin–aldosterone system activation.

Postpartum Page kidney secondary to HELLP (hemolysis, elevated liver enzyme, low platelets) syndrome has also been described; the authors in the reported case hypothesize that the spontaneous gradual development of a right renal subcapsular hematoma in the immediate postpartum period may have evolved^10^. To add, Baishya et al^11^ proposed that hypertension might be one of the causes of Page kidney. However, the Page kidney can develop remotely after the causative event, and also the history of trauma may not always be obvious^1^.

The presentation of Page kidney may range from nonspecific clinical presentation with an insidious onset, to a catastrophic event of hypertensive urgency or emergency^12^. Flank hematoma can be seen in recent perinephric bleeding. Often physical examination fails to reveal anything except for hypertension. It is important to note that a significant time interval could be present between trauma and the diagnosis of hypertension. The interval could be a few days but can be decades long and well-forgotten as well^1^. Our patient had neither a history of traumas nor known systemic diseases. Besides, all coagulative parameters were within normal limits. The subcapsular hematomas developed idiopathically in both kidneys. The antenatal rise in BP and subtle trauma could be the potential risk factors.

Ultrasonography and computed tomography of the kidneys are the most frequently used for diagnosing a Page kidney. Ultrasound is cheap, easily available, and noninvasive, quick, easy to perform, however, can sometimes miss small subcapsular hematoma and thus have to be confirmed with a CT scan. CT scan of the abdomen has the benefit of being able to detect small hematomas as well as identification of an underlying mass if any and is most frequently used^13^,^14^.

Workup for neoplastic lesions and coagulation disorders from an important part of the diagnostic workup in cases of spontaneous renal hemorrhage^15^. Our patient was thoroughly evaluated for the cause, however, no etiology was found.

Management strategies in Page kidney are directed at controlling the rise in BP. Though no standardized treatment exists for the Page kidney due to subcapsular hematoma, medical, and invasive treatment modalities have been tried successfully. Medical management with the use of antihypertensive and regular follow-ups form the first line of treatment in Page kidneys. As secondary hypertension results from the vasculature’s response to angiotensin-I and the effect of aldosterone on the renal collecting system, treatment with angiotensin-converting enzyme inhibitors or aldosterone receptor blockers is typically effective at counteracting renin’s downstream effects, given the cautious use of angiotensin-converting enzyme inhibitor in the setting of acute kidney injury^16^.

The age of the hematoma is an important consideration for the choice of treatment. Often hematomas less than 3 weeks tend to resolve spontaneously and are treated medically with close observation. However, with uncontrolled/poorly controlled hypertension despite medical therapy, the hematoma should be drained. In addition, interventional procedures may be needed for larger, symptomatic, deteriorating renal function, or enlarging collections^17^. Percutaneous drainage, surgical decortication, laparoscopic intervention, and nephrectomy are necessary in cases of organized late hematomas^18^. It has been shown by recent case reports that minimally invasive procedures, for instance, laparoscopy-assisted or radiology-assisted percutaneous drainage are viable therapeutic alternatives. Percutaneous drainage has been observed to have better success rates in subcapsular hematomas less than 3 weeks. However, more chronic organized hematomas frequently require invasive procedures for sufficient evacuation^19^.

Medical treatment of hypertension may be required for a short period if the cause of renal compression resolves spontaneously or the following intervention. Despite adequate treatment, a significant proportion of patients with Page kidney can develop chronic hypertension that mandates regular BP surveillance^7^.

The patient described in this case was initially managed with aldosterone receptor blocker which could not decrease her BP to an optimal level which succeeded after percutaneous drainage of the hematoma. She is under regular follow-up.

## Conclusion

Spontaneous bilateral Page kidney is a rare but potentially treatable and curable form of hypertension. Ultrasound and CT scans are easy and effective methods for the early diagnosis of this disorder. Percutaneous drainage is an effective method to drain the hematoma which can improve hypertension.

## Ethical approval

Not required.

## Patient consent

Written informed consent was obtained from the patient for publication of this case report and accompanying images. A copy of the written consent is available for review by the Editor-in-Chief of this journal on request.

## Sources of funding

This research work did not receive any kind of funding.

## Authors’ contribution

S.S., P.R., and E.P. were involved in the study concept, data collection, and writing of the manuscript. P.R. and D.S. were involved in the treatment and reviewing of the manuscript. All the authors were involved in the final review of the manuscript.

## Conflicts of interest disclosure

None to declare.

## Research registration unique identifying number (UIN)

None.

## Guarantor

Dr. Suraj Shrestha. E-mail: multisurazz@gmail.com

## Provenance and peer review

Not commissioned, externally peer-reviewed.

## References

[1] Vaidya PN Rathi BM Finnigan NA . Page kidney. StatPearls. StatPearls Publishing; 2022.

[2] Diamond JA . Hypertension due to perinephric compression: the ‘Page’ kidney. Am J Hypertens 2001;14:305. 11281245 10.1016/s0895-7061(01)01286-9

[3] Mufarrij P Sandhu JS Coll DM . Page kidney as a complication of percutaneous antegrade endopyelotomy. Urology 2005;65:592. 10.1016/j.urology.2004.09.047 15780391

[4] Arslan S . Bilateral nontraumatic recurrent Page kidney. Radiol Case Rep 2017;12:511–3. 28828114 10.1016/j.radcr.2017.05.003 PMC5552010

[5] Warnich I Nicolaou M Sofianos Z . Page kidney: a rare cause of secondary hypertension. SA J Radiol 2019;23:1762. 31754544 10.4102/sajr.v23i1.1762 PMC6837813

[6] Capitanini A Tavolaro A Rosellini M . Wunderlich syndrome during antiplatelet drug therapy. Clin Nephrol 2009;71:342–4. 19281750 10.5414/cnp71342

[7] Smyth A Collins CS Thorsteinsdottir B . Page kidney: etiology, renal function outcomes and risk for future hypertension. J Clin Hypertens 2012;14:216–221. 10.1111/j.1751-7176.2012.00601.x PMC8108801 22458742

[8] Cozzoli A Teppa A Gregorini G . Spontaneous renal hemorrhage occurring during pregnancy. J Nephrol 2003;16:595. 14696765

[9] Agha RA Franchi T Sohrabi C . SCARE Group. The SCARE 2020 Guideline: Updating Consensus Surgical CAse REport (SCARE) Guidelines. Int J Surg 2020;84:226–30. 33181358 10.1016/j.ijsu.2020.10.034

[10] Kamath SU Patil B Patwardhan SK . Postpartum page kidney secondary to HELLP syndrome. J Clin Diagn Res 2018;12:5–6.

[11] Baishya RK Dhawan DR Sabnis RB . Spontaneous subcapsular renal hematoma: a case report and review of literature. Urol Ann 2011;3:44–6. 21346835 10.4103/0974-7796.75852 PMC3037002

[12] Kenis I Werner M Nacasch N . Recurrent non-traumatic page kidney. Isr Med Assoc J 2012;14:452–3. 22953625

[13] Kiczek M Udayasankar U . Page kidney. J Urol 2015;194:1109–10. 26173105 10.1016/j.juro.2015.07.035

[14] Zhang JQ Fielding JR Zou KH . Etiology of spontaneous perirenal hemorrhage: a meta-analysis. J Urol 2002;167:1593–6. 11912370 10.1097/00005392-200204000-00006

[15] Ahn T Roberts MJ Navaratnam A . Recurrent spontaneous renal haemorrhage due to polyarteritis nodosa: a medical cause for a surgical problem. ANZ J Surg 2018;88:1347–8. 28239941 10.1111/ans.13914

[16] Wahdat R Schwartz C Espinosa J . Page kidney: taking a page from history. Am J Emerg Med 2017;35:193.e1–e2. 10.1016/j.ajem.2016.06.095 27439385

[17] Santucci RA Wessells H Bartsch G . Evaluation and management of renal injuries: consensus statement of the renal trauma subcommittee. BJU Int 2004;93:937–54. 15142141 10.1111/j.1464-4096.2004.04820.x

[18] Babel N Sakpal SV Chamberlain RS . The Page kidney phenomenon secondary to a traumatic fall. Eur J Emerg Med 2010;17:24–6. 19543099 10.1097/MEJ.0b013e32832ce8ba

[19] Kobel MC Nielsen TK Graumann O . Acute renal failure and arterial hypertension due to subcapsular haematoma: is percutaneous drainage a feasible treatment? BMJ Case Rep 2016;2016:bcr2015212769. 10.1136/bcr-2015-212769 PMC4735349 26783007

